# Association of cancer diagnosis with disability status among older survivors of colorectal cancer: a population-based retrospective cohort study

**DOI:** 10.3389/fonc.2024.1283252

**Published:** 2024-03-15

**Authors:** Shiming Zhang, Lin-Na Chou, Michael D. Swartz, Hemalkumar B. Mehta, James S. Goodwin, Yong-Fang Kuo, Sharon Hermes Giordano, Carole A. Tucker, Karen M. Basen-Engquist, Elizabeth J. Lyons, Brian Downer, Susan K. Peterson, Tru Cao, Maria C. Swartz

**Affiliations:** ^1^ Department of Biostatistics and Data Science, The University of Texas Health Science Center at Houston School of Public Health, Houston, TX, United States; ^2^ Division of Pediatrics, The University of Texas MD Anderson Cancer Center, Houston, TX, United States; ^3^ Department of Biostatistics and Data Science, The University of Texas Medical Branch, Galveston, TX, United States; ^4^ Department of Epidemiology, John Hopkins Bloomberg School of Public Health, Baltimore, MD, United States; ^5^ Department of Internal Medicine, The University of Texas Medical Branch, Galveston, TX, United States; ^6^ Department of Health Services Research, The University of Texas MD Anderson Cancer Center, Houston, TX, United States; ^7^ Department of Physical Therapy, The University of Texas Medical Branch, Galveston, TX, United States; ^8^ Department of Health Disparities Research, The University of Texas MD Anderson Cancer Center, Houston, TX, United States; ^9^ Department of Nutrition, Metabolism and Rehabilitation Sciences, The University of Texas Medical Branch, Galveston, TX, United States; ^10^ Department of Population Health and Health Disparities, The University of Texas Medical Branch, Galveston, TX, United States; ^11^ Department of Behavioral Science, The University of Texas MD Anderson Cancer Center, Houston, TX, United States

**Keywords:** colorectal neoplasms, cancer survivors, aged, mobility limitations, risk factors

## Abstract

**Background:**

Older cancer survivors likely experience physical function limitations due to cancer and its treatments, leading to disability and early mortality. Existing studies have focused on factors associated with surgical complications and mortality risk rather than factors associated with the development of poor disability status (DS), a proxy measure of poor performance status, in cancer survivors. We aimed to identify factors associated with the development of poor DS among older survivors of colorectal cancer (CRC) and compare poor DS rates to an age-sex-matched, non-cancer cohort.

**Methods:**

This retrospective cohort study utilized administrative data from the Texas Cancer Registry Medicare-linked database. The study cohort consisted of 13,229 survivors of CRC diagnosed between 2005 and 2013 and an age-sex-matched, non-cancer cohort of 13,225 beneficiaries. The primary outcome was poor DS, determined by Davidoff’s method, using predictors from 12 months of Medicare claims after cancer diagnosis. Multivariable Cox proportional hazards regression was used to identify risk factors associated with the development of poor DS.

**Results:**

Among the survivors of CRC, 97% were 65 years or older. After a 9-year follow-up, 54% of survivors of CRC developed poor DS. Significant factors associated with future poor DS included: age at diagnosis (hazard ratio [HR] = 3.50 for >80 years old), female sex (HR = 1.50), race/ethnicity (HR = 1.34 for Hispanic and 1.21 for Black), stage at diagnosis (HR = 2.26 for distant metastasis), comorbidity index (HR = 2.18 for >1), and radiation therapy (HR = 1.21). Having cancer (HR = 1.07) was significantly associated with developing poor DS in the pooled cohorts; age and race/ethnicity were also significant factors.

**Conclusions:**

Our findings suggest that a CRC diagnosis is independently associated with a small increase in the risk of developing poor DS after accounting for other known factors. The study identified risk factors for developing poor DS in CRC survivors, including Hispanic and Black race/ethnicity, age, sex, histologic stage, and comorbidities. These findings underscore the importance of consistent physical function assessments, particularly among subsets of older survivors of CRC who are at higher risk of disability, to prevent developing poor DS.

## 1 Introduction

Colorectal cancer (CRC) is the third most common cancer in the United States (1), impacting over 1 million individuals, with 73% aged 65 years and older (2, 3). Given the projection that three-fourths of cancer survivors will be 65 years and older by 2040 (4), caring for this growing population will challenge the health care system as they are vulnerable to conditions such as premature death and decline in physical function earlier than expected based on their biological age (5, 6).

Cancer and cancer-related therapies can negatively impact physical function, resulting in disability, loss of independence, and early mortality (3, 7, 8). In fact, a recent cohort study showed that survivors of CRC who reported functional decline had a 55% higher risk of death than those without functional decline (9). Despite these findings, research on functional impairments of adult cancer survivors (10, 11), as well as identifying factors associated with the development of disabilities in cancer survivors, remain limited (12). Furthermore, the utilization of rehabilitation services among cancer survivors with physical limitations remains low, with as few as 2% receiving such services (13). Consequently, there is a significant gap in adequately addressing disabilities within this population.

While most risk prediction models focus on mortality risks (14, 15) and surgical complications (16–19), few studies have evaluated the potential loss of functional independence among older survivors of cancer, including survivors of CRC. Previous studies conducted during and after cancer treatment have found associations between sociodemographic, health, and clinical factors with poor health and poor disability status among cancer patients, including those diagnosed with CRC (20–22). However, these studies primarily relied on self-reported methods, which may be vulnerable to potential biases and inaccuracies due to low response rates (23) and increased patient burden (24). Furthermore, prior research did not examine risk factors associated with the development of poor disability status, a proxy measure of Eastern Cooperative Oncology Group Performance Status (ECOG PS) of 3 or 4 (25), among cancer survivors or compare poor disability status rates between the cancer survivor population and non-cancer populations; instead, they only assessed patients’ status during treatment and after diagnosis (26). Therefore, it remains unclear what the physical function status of patients was before a cancer diagnosis. The factors contributing to the development of poor disability status in cancer survivors, and how survivors’ rates of development of poor disability status compared to the non-cancer population, remained unknown.

To better understand the impact of risk factors contributing to the development of poor disability status among cancer survivors, it is important to study long-term functional outcomes using methods beyond self-report. However, the systematic evaluation of functional levels before and during cancer treatment is limited, hindering the estimation of impairment burden at the population level and guiding clinical practice amid rapid treatment changes (27). To address the aforementioned gaps, this study aimed to identify the association between demographic variables, comorbid health problems, and cancer-related clinical characteristics with future disability status among older survivors of CRC after diagnosis, and to compare disability status between CRC survivors and a non-cancer cohort. Identifying these risk factors can contributes to the literature, enabling the development of mitigation plans to prevent or slow down physical function decline, potentially improving quality of life. This may also allow identifying high-risk patient subgroups for targeted intervention development.

## 2 Methods

### 2.1 Data source

We conducted a retrospective cohort study using the Texas Cancer Registry (TCR) Medicare-linked database as our data source. Previous publications (28–30) provide more details about the database. Briefly, the TCR, supported by the Surveillance, Epidemiology, and End Results (SEER) Program, is a statewide and population-based cancer registry that contains cancer diagnosis information, such as cancer diagnosis time, cancer type, and histologic cancer stage. Approximately 95% of the older patients in the TCR, defined as 65 years and older, could be linked to Medicare (30, 31).

The TCR Medicare-linked database covers the period from 2004 to 2014 includes two populations: individuals with a cancer diagnosis between 1995 and 2013 and a randomly selected 5% sample of non-cancer Medicare beneficiaries. The Patient Entitlement and Diagnosis Summary File (PEDSF) was used for individuals with cancer, and the Summarized Denominator File (SUMDENOM) was used for individuals without cancer to determine demographic factors and Medicare enrollment status. Information related to cancer diagnosis, such as diagnosis time, cancer type, and secondary cancer, was obtained from PEDSF. Medicare inpatient and outpatient claims, including Medicare Provider Analysis and Review (MedPAR) files, Outpatient Standard Analytic files (OUTSAF), carrier files, durable medical equipment (DME) files, and hospice files during 2004 and 2014 were used to determine cancer treatment, comorbidity, and disability status (32). All data were de-identified, and no protected health information was shared with the analytical team. The study was approved by the institutional review board at The University of Texas Medical Branch.

### 2.2 Study cohorts

The study comprised two cohorts: the CRC patient cohort and a matched cohort of non-cancer Medicare beneficiaries. In both cohorts, 97.2% were aged 65 years or older.

Cancer patients were included in our study if they were diagnosed with CRC as the primary cancer between 2005 and 2013, with no secondary or other cancer diagnosis (i.e., any type of non-CRC cancers) within five years after the primary cancer diagnosis. A CRC diagnosis was determined by the International Classification of Disease for Oncology, Third Edition (ICD-O-3) codes C180, C182-C189, C199, C209 (33, 34). Additionally, to be included in our study, patients must be continuously enrolled in Medicare fee-for-service for 12 months before and after the primary cancer diagnosis (**Table 1**). To observe the development of disability among older survivors of CRC, which was the primary outcome of interest, we excluded individuals who: (1) were listed as deceased in TCR, (2) enrolled in Medicare due to disability, or (3) had a current disability (Current reason for Medicare entitlement is disability OR algorithm-defined poor disability status prior to cancer diagnosis (25)). For a more detailed view of how we applied the inclusion and exclusion criteria for our study, see **Table 1**. Our final cancer cohort consisted of 13329 patients.

**Table 1:** Flowchart of Cohort Selection for Colorectal Cancer Survivors.

Selection	Criteria	No. of Eligible Subjects
Step 1	Primary diagnosis as colorectal cancer patients	116962
Step 2	Only one primary CRC, or only CRC within 5 years	101233
Step 3	Diagnosed during 2005-2013 (Time of diagnosis between 01/2005 and 12/2013)	44508
Step 4	Exclude source of TCR reporting is autopsy or death certificate	43278
Step 5	Exclude subjects with the original reason for Medicare entitlement as disability	34681
Step 6	Exclude subjects with a current reason for Medicare entitlement as disability	34678
Step 7	12 months of continuous Medicare enrollment (part A and B, no HMO) before diagnosis	20258
Step 8	12 months of continuous Medicare enrollment (part A and B, no HMO) after diagnosis	14508
Step 9	Exclude subjects with algorithm-defined disability in the year prior to cancer diagnosis	13229

Once we selected the cancer survivors for our study, we selected the non-cancer cohort from the TCR-Medicare linked database who were Medicare beneficiaries without cancer and without entitlement to disability benefits. Initially, a pool of non-cancer patients was created by randomly selecting 5% of Medicare beneficiaries from a non-cancer control population who were also Medicare beneficiaries but did not have entitlement to disability benefits. An individual exact matching procedure was applied. First, a subject was randomly selected from the CRC cohort of 13,229 subjects without replacement. To match with the selected CRC patient on age, the CRC diagnosis date of the selected subject was applied to the non-cancer cohort as the index date. Non-cancer patients needed to satisfy three eligibility criteria: age at index date, sex, and 12 months of continuous Medicare enrollment before the index date. After identifying qualified matching non-cancer subjects, one subject was randomly selected into the non-cancer group. This process was repeated 13,229 times until the last survivors of CRC were selected. As a result, 13,225 survivors of CRC were successfully matched to non-cancer subjects, while four cancer survivors could not be matched with non-cancer patients meeting the eligibility criteria.

### 2.3 Outcome

The study’s primary outcome was disability status (good/poor),which was identified using Davidoff’s method (25). This is a claims-based prediction model-derived disability status measure. Davidoff’s method is a validated multivariate, claims-based prediction model that has shown good performance in both estimation and validation samples in predicting disabilities (25). Details of the development of the claims-based prediction model derived disability status measure have been published elsewhere (25). Briefly, the disability status model was derived using claims-based predictors to predict poor disability status defined by survey-based performance status metrics that aligned with poor ECOG PS (25), and validated in four cohorts of cancer patients (32). Specifically, the claims-based prediction model derived disability status included indicators for health care services that were expected to differ based on the disability status. The claims files used for Davidoff’s method included the national claims history (NCH) claims data, DME claims data, Hospice claims data, and patient demographics. The predictors used for Davidoff’s model were organized in the following categories: evaluation and management/other visits, minor procedures, ambulatory procedures, preventive services, major procedures, durable medical equipment, imaging, and others (25). The information is then used to predict a disability status probability that ranges from 0 to 1 with 0.11 used as the threshold to assign a disability status indicator equal to 1 denoting poor disability status. (25). The predicted disability status was measured in the first year of cancer diagnosis and then was reassessed over a 12-month period every month throughout the follow-up period (Month 0 [cancer diagnosis] to Month 12, Month 1 to Month 13, Month 2 to Month 14, etc.) The study included data through December 2014, and the longest study follow-up time was 119 months.

### 2.4 Covariates

We have selected the following covariates based on the previous studies (20–22) that examined these covariates’ relation with poor health and disability status among cancer patients, including those diagnosed with CRC. Demographic factors (age, sex, race/ethnicity, and reason for Medicare entitlement) and resident location (ZIP code) were derived from the PEDSF for cancer patients and the SUMDENOM for individuals without cancer. ZIP code data were linked with American Community Survey data to determine the community-level socioeconomic status (education and income). For clinical characteristics, the comorbidity score was based on the Klabunde modification claims-based algorithm of the Charlson comorbidity index (CCI; 35), which was measured at baseline (1 year before index date) and categorized into three groups (0, 1, or ≥2). For other clinical variables seen only in the CRC survivor cohort, the histologic cancer stage was determined by the PEDSF record, and the cancer-related treatment (chemotherapy, radiation therapy, and surgery) was determined by the ICD-9-CM diagnostic code, ICD-9-CM procedure code, Current Procedural Terminology code, and revenue center code in Medicare claims (**Table S1**). Medical claims (MedPAR, OUTSAF, and carrier files) were used to determine the cancer-related treatment in the first year after cancer diagnosis and during the follow-up period after the cancer diagnosis up to 119 months (32).

Age, sex, race/ethnicity, ZIP code-level house income, ZIP code-level education level, and CCI were used for both the cancer and non-cancer cohorts. Cancer stage (histologic stage and American Joint Committee on Cancer [AJCC] stage) at diagnosis and Medicare claims defined cancer-related treatment (surgery, radiation, and chemotherapy status) were used to investigate the influence of demographics, cancer status, and cancer treatment on disability among survivors of CRC. Race/ethnicity, income, education, and CCI also were used as confounders for estimating the influence of cancer on disability development.

### 2.5 Statistical analysis

The descriptive statistics consisted of the mean and standard deviation for continuous variables and a frequency count and percentage for categorical variables. To measure the time to the development of poor disability status, the Kaplan-Meier estimate was used to evaluate the fraction of patients’ functional physical condition for the length of time after treatment or surviving cancer (36). Log-rank tests were then applied to compare the time to develop poor disability status among different categories of demographic factors or clinical characteristics for survivors of CRC. Point estimates and 95% confidence intervals (CIs) were presented at three time points (3, 5, and 9 years). Those three specific time points after cancer diagnosis were considered for observing poor disability status because each represents an important milestone in the cancer survivorship journey (the most recommended follow-up time of reoccurrence, and the lower and upper bound of post-diagnosis time of long-term survivors of CRC (37). According to Figueredo et al. (38) and Thong et al. (39), these time points are significant for monitoring the health and well-being of cancer survivors (38, 39), and the changes in the surveillance plan typically take place at around 3 years, 5 years, and 9 years following completion of treatment (40).

To identify the factors associated with the risk of developing poor disability status, multivariable Cox proportional hazards regressions were used with an adjustment for all covariates and censored events, including death, Medicare discontinuation, and the end of the study. Hazard ratios (HRs) and 95% CIs were estimated to evaluate the associations between each attribute with poor disability status. For the comparison between the cancer cohort and the matched non-cancer cohort, the marginal approach Cox model was applied to account for the intracluster dependence from the matching design (41, 42). A time-dependent covariate Cox model was included as a sensitivity analysis to include cancer treatment as a time-varied covariate among the cancer cohort. Assumptions of the Cox model were examined through the Kaplan–Meier curves, and there was no significant proportional hazards violation for each predictor according to the comparisons of the Nelson-Aalen estimate of cumulative hazard functions All statistical analyses and figure generations were performed with SAS 9.4 (SAS Institute Inc, Cary, NC).

## 3 Results

For the CRC patient cohort, the mean age at diagnosis (Time 0) was 75.6 years (SD 6.94); 51% of the study sample were female, most whom were non-Hispanic White (**Table 2**). At Time 0, when cancer was diagnosed, more than half the survivors did not have a comorbidity, and 46% had localized disease on histologic staging. During the first year after cancer diagnosis, most patients (90%) had undergone surgery, 14% had received radiation therapy, and 32% had received chemotherapy.

**Table 2:** Participant Characteristics (*N* = 13,229).

Variable	No. (%)
	Colorectal cancer
Age at diagnosis, years	
Mean (SD)	75.6 (6.94)
≤70	3702 (27.98%)
71–75	3327 (25.15%)
76–80	2889 (21.84%)
>80	3311 (25.03%)
Sex	
Male	6433 (48.63%)
Female	6796 (51.37%)
Origin and race/ethnicity^‡^	
Hispanic	1891 (14.29%)
White	10170 (76.88%)
Black	897 (6.78%)
Other	271 (2.05%)
Income^§^	
Q1 (< $39,350 per year)	3335 (26.61%)
Q2 ($39,350 - $47,398 per year)	3109 (24.81%)
Q3 ($47,399 - $60,680 per year)	2994 (23.89%)
Q4 (>$60,680 per year)	3095 (24.69%)
Education (percent of people without high school diploma)^§^	
Q1 (≥ 23.2%)	3768 (30.03%)
Q2 (≥ 15.7%, < 23.2%)	3347 (26.67%)
Q3 (≥ 9.3%, < 15.7%)	2614 (20.83%)
Q4 (< 9.3%)	2819 (22.47%)
Charlson comorbidity index	
Mean (SD)	0.8 (1.23)
No	7153 (54.07%)
At least 1	6076 (45.93%)
Cancer histologic stage	
In situ	506 (3.82%)
Localized	6097 (46.09%)
Regional	4316 (32.63%)
Distant	1342 (10.14%)
Unstaged	968 (7.32%)
AJCC Stage 6th	
Stage 0	883 (6.67%)
Stage I	3132 (23.68%)
Stage II	3294 (24.90%)
Stage III	2921 (22.08%)
Stage IV	1208 (9.13%)
Unstaged	1791 (13.54%)
Surgery	
No	1314 (9.93%)
Yes	11915 (90.07%)
Radiation	
No	11382 (86.04%)
Yes	1847 (13.96%)
Chemotherapy	
No	9041 (68.34%)
Yes	4188 (31.66%)

^‡^ Origin recode NHIA was applied to define Hispanic group.

^§^Quartile was based on distribution of all covered zip code. There were some patients without data.

**Figure 1:**
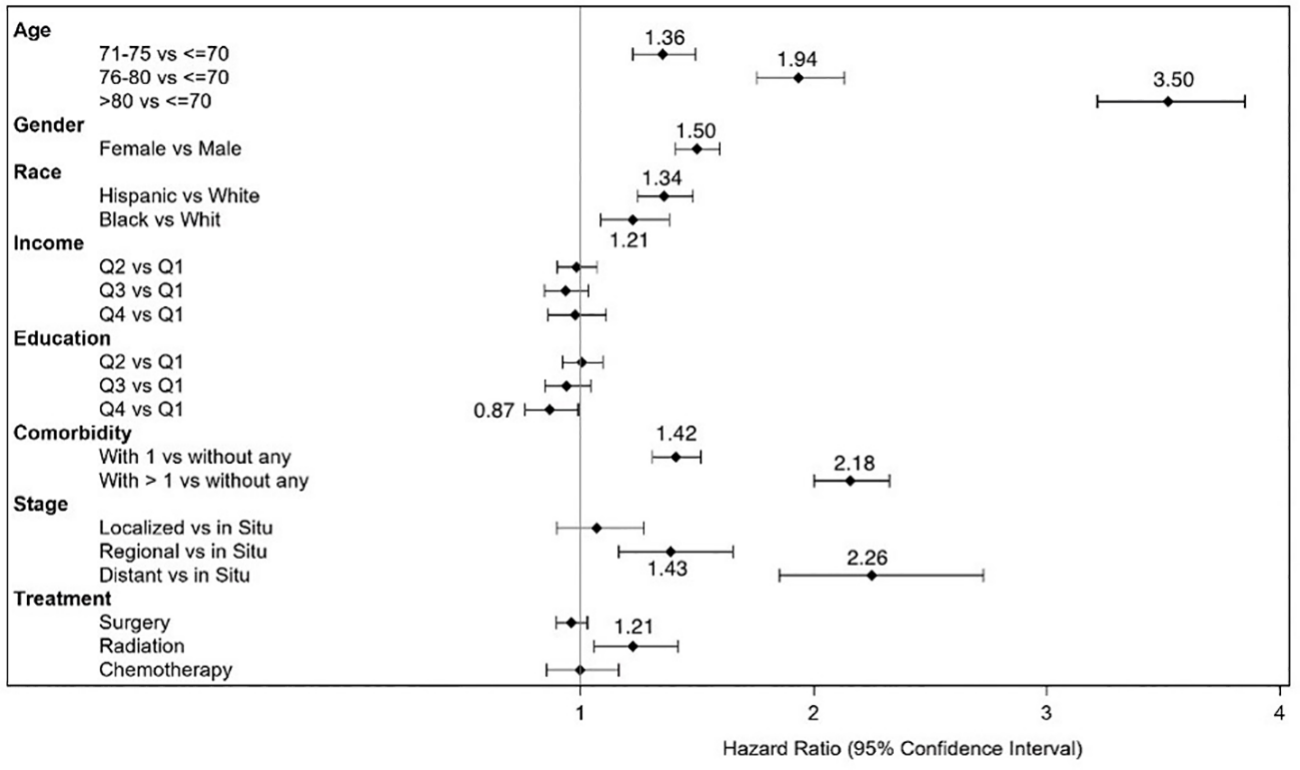
Forest plot of hazard ratios and 95% confidence intervals for development of disability status in survivors of colorectal cancer.

**Table 3** shows that overall, within the CRC survivors, about half (54%) developed poor disability status within 9 years of cancer diagnosis. The log-rank test results in **Table 3** indicated significant differences in the disability rates and their 95% conference intervals at different levels of age, sex, cancer histologic stage, AJCC stage, CCI, surgery status, and chemotherapy status but not for radiation therapy status. For example, for histologic and AJCC stages I and higher, the disability rates increased with stage. Further, time since diagnosis at 3, 5, and 9 years after diagnosis was highly associated with the development of poor disability status. **Figure 1** presents a forest plot of HRs and 95% CIs obtained from the multivariable Cox regression model, illustrating the relationship with poor disability status among survivors of CRC. Factors that were significantly associated with an increased risk of poor disability status in the CRC survivor cohort were older age [HR (95% CI) = 3.50 (3.19–3.83) for >80 years old], female sex [HR (95% CI) = 1.50 (1.41–1.60)], race/ethnicity [HR (95% CI) = 1.34 (1.22–1.46) for Hispanic and 1.21 (1.07–1.36) for Black], stage at diagnosis [HR (95% CI) = 2.26 (1.85–2.76) for distant stage], comorbidity [HR (95% CI) = 1.42 (1.32–1.53) for one comorbidity and HR (95% CI) = 2.18 (2.02–2.35) for more than one], and ever had radiation [HR (95% CI) = 1.21 (1.10–1.33)]. We conducted an additional analysis whereby we examined the treatment variable as a time-dependent variable. Being in older age groups, female sex, Hispanic or Black race/ethnicity, having one or more comorbidities, having regional and distant disease, and having radiation therapy remained risk factors for the development of poor disability status (**Supplementary Table S2**).

**Table 3:** Disability Rate at 3, 5, and 9 Years after Cancer Diagnosis.

Category	Disability Rate (95% CI)			
	3 years	5 years	9 years	*p-*value
Colorectal cancer	0.27 (0.27–0.28)	0.37 (0.36–0.38)	0.54 (0.53–0.56)	
Age at diagnosis, years				<0.001
≤70	0.15 (0.14–0.17)	0.22 (0.21–0.24)	0.36 (0.33–0.39)	
71–75	0.21 (0.19–0.22)	0.30 (0.28–0.32)	0.47 (0.43–0.50)	
76–80	0.30 (0.28–0.32)	0.40 (0.38–0.42)	0.63 (0.59–0.66)	
>80	0.46 (0.44–0.47)	0.59 (0.57–0.61)	0.78 (0.75–0.81)	
Sex				<0.001
Male	0.22 (0.21–0.23)	0.30 (0.29–0.32)	0.46 (0.44–0.49)	
Female	0.33 (0.32–0.34)	0.43 (0.42–0.45)	0.61 (0.59–0.63)	
Race/ethnicity^‡^				<0.001
Hispanic	0.34 (0.32–0.37)	0.45 (0.42–0.47)	0.61 (0.56–0.65)	
White	0.26 (0.25–0.27)	0.35 (0.34–0.36)	0.53 (0.51–0.55)	
Black	0.33 (0.30–0.37)	0.43 (0.39–0.47)	0.60 (0.53–0.66)	
Other	0.29 (0.23–0.34)	0.33 (0.27–0.40)	0.54 (0.43–0.66)	
Histologic stage^§^				<0.001
*In situ*	0.21 (0.17–0.25)	0.28 (0.24–0.33)	0.45 (0.38–0.53)	
Localized	0.23 (0.22–0.24)	0.33 (0.31–0.34)	0.51 (0.49–0.53)	
Regional	0.30 (0.29–0.32)	0.40 (0.39–0.42)	0.57 (0.54–0.60)	
Distant	0.40 (0.37–0.43)	0.54 (0.49–0.58)	0.68 (0.61–0.75)	
AJCC stage (6th edition)^§^				<0.001
Stage 0	0.21 (0.18–0.24)	0.30 (0.26–0.33)	0.46 (0.41–0.51)	
Stage I	0.21 (0.19–0.22)	0.30 (0.28–0.32)	0.49 (0.46–0.52)	
Stage II	0.28 (0.26–0.30)	0.38 (0.36–0.40)	0.56 (0.53–0.59)	
Stage III	0.31 (0.29–0.32)	0.40 (0.38–0.43)	0.57 (0.53–0.60)	
Stage IV	0.40 (0.36–0.43)	0.56 (0.51–0.61)	0.67 (0.59–0.74)	
Charlson comorbidity index				<0.001
No	0.21 (0.20–0.22)	0.30 (0.29–0.31)	0.46 (0.44–0.48)	
At least 1	0.35 (0.33–0.36)	0.46 (0.45–0.48)	0.66 (0.63–0.68)	
Surgery				<0.001
No	0.37 (0.34–0.40)	0.47 (0.44–0.51)	0.58 (0.53–0.63)	
Yes	0.27 (0.26–0.27)	0.36 (0.35–0.37)	0.54 (0.52–0.56)	
Radiation				0.364
No	0.27 (0.26–0.28)	0.37 (0.36–0.38)	0.54 (0.53–0.56)	
Yes	0.28 (0.26–0.31)	0.39 (0.36–0.41)	0.54 (0.50–0.58)	
Chemotherapy				0.001
No	0.28 (0.27–0.29)	0.38 (0.37–0.39)	0.56 (0.54–0.58)	
Yes	0.26 (0.25–0.28)	0.35 (0.33–0.37)	0.51 (0.48–0.54)	

^‡^ Origin recode NHIA was applied to define the Hispanic group.

^§^ Patients with un-staged were not included.

We compared the development of poor disability status among the cancer-free cohort to that of the CRC cohort. **Table 4** compares the baseline characteristics of these two matched (by age and sex) cohorts. Although race/ethnicity, income, and education characteristics were significantly different between the CRC cohort and the non-cancer cohort, most likely due to the large sample size of each cohort, the distributions of these variables between the two cohorts were not clinically dissimilar. The number of comorbidities, however, varied significantly and substantially between the two cohorts. In the matched non-cancer cohort, a large percentage of Medicare beneficiaries had no comorbidities at baseline.

**Table 4:** Comparison of Baseline Characteristics between Matched Cancer Patient Cohort and Non-Cancer Cohort.

Variable	No. (%)		*p*-value^¥^
	Colorectal cancer group (*N* = 13,225)	Non-cancer group (*N* = 13,225)	
Age at index date, years			
Mean (SD)	75.6 (6.92)	75.6 (6.92)	1.000
≤70	3700 (27.98%)	3700 (27.98%)	1.000
71-75	3327 (25.16%)	3327 (25.16%)	
76-80	2889 (21.84%)	2889 (21.84%)	
>80	3309 (25.02%)	3309 (25.02%)	
Sex			1.000
Male	6429 (48.61%)	6429 (48.61%)	
Female	6796 (51.39%)	6796 (51.39%)	
Race/ethnicity^‡^			<0.001
Hispanic	432 (3.27%)	682 (5.16%)	
White	11529 (87.18%)	11293 (85.39%)	
Black	964 (7.29%)	846 (6.40%)	
Other	285 (2.16%)	383 (2.90%)	
Unknown	15 (0.11%)	21 (0.16%)	
Income^§^			<0.001
Q1 (low)	3335 (26.62%)	3394 (26.53%)	
Q2	3109 (24.81%)	2915 (22.78%)	
Q3	2992 (23.88%)	2995 (23.41%)	
Q4 (high)	3093 (24.69%)	3491 (27.28%)	
Education^§^			<0.001
Q1 (low)	3767 (30.03%)	3639 (28.37%)	
Q2	3347 (26.68%)	3055 (23.82%)	
Q3	2613 (20.83%)	2741 (21.37%)	
Q4 (high)	2817 (22.46%)	3390 (26.43%)	
Charlson comorbidity index			
Mean (SD)	0.8 (1.23)	0.3 (0.84)	<0.001
No	7153 (54.09%)	11046 (83.52%)	<0.001
At least 1	6072 (45.91%)	2179 (16.48%)	

^‡^ Medicare race.

^§^Quartile was based on the distribution of all covered zip codes. There were some patients without data.

^¥^ Chi-square test was applied for categorical variable, and t-test was applied for continuous variable.

As seen in **Table 5**, the probability of disability at three different time points was higher for the CRC survivor cohort than for the non-cancer cohort. Further, half the CRC survivors developed poor disability status within 9 years after diagnosis. In contrast, less than half the age-sex-matched non-cancer cohort developed poor disability status after 9 years of follow-up (log-rank test, *p* < 0.01). Additionally, the HRs and 95% CIs for the matched cancer and non-cancer cohorts from the Cox regression model indicated that having a cancer diagnosis (HR [95% CI] = 1.07 [1.02–1.13]) was associated with the development of poor disability status after adjustment for age, sex, race, income, education, and comorbidity (**Supplementary Table S3**).

**Table 5:** Disability Rate at Different Time Points, Median Time to Develop Disability status, and Adjusted HR for Developing disability status (*N* = 13,225 matched pairs).

Cohort	Disability rate (95% CI)			Median year (95% CI)	aHR^*^ (95% CI) p-value^†^
	3 Years	5 Years	9 Years		
Colorectal cancer	0.27 (0.2–0.28)	0.37 (0.36–0.38)	0.54 (0.53–0.56)	7.92 (7.58–8.33)	1.07 (1.02–1.13)
Non–cancer	0.21 (0.20–0.22)	0.32 (0.31–0.33)	0.48 (0.46–0.49)	>9 years**^‡^**	<0.01

*aHR: Adjusted hazard ratio was estimated by adjusting age, sex, race, income, education, and baseline comorbidity.

†Log-rank test p-value.

‡ The median time to develop disability status was longer than the 9 years of study follow-up due to the fact that only 48% of non-cancer subjects developed poor disability status by the end of extracted follow-up time.

## 4 Discussion

Currently, limited studies have evaluated the development of poor disability status and factors associated with the development of poor disability status, because existing models either focus on evaluating mortality and surgical complications or identifying risk factors associated with poor health and existing poor disability status. To address the gap in the literature, we used a claims-based prediction model-derived disability status measure to systematically evaluate the functional levels, before, during, and after cancer treatment, and risk factors associated with the development of poor disability status. The results of this large retrospective study provided evidence that, within a CRC cohort, factors significantly associated with the risk of developing poor disability status include older age (>80 years), female sex at birth, Hispanic or Black ethnicity, having histologic findings of regional or distant disease, AJCC stage III/IV CRC diagnosis, having more than one comorbidity, and receiving radiation therapy. After combining the CRC cohort with the matched non-cancer cohort, a CRC diagnosis was associated with a small increase in the risk of developing poor disability status [HR (95% CI) = 1.07 (1.02–1.13)]. Although our HR of 1.07 is small, it remains significant in line with other disability research (43). Notably, a recent meta-analysis on disability reported a risk ratio of 1.07, a similar risk ratio magnitude, has been associated with an incident of disability with each one-second increase in the chair rise test, as highlighted by Braun et al. (43) (43). Hazard ratios and risk ratios of this magnitude are essentially equivalent, especially when the probability of the event—in this case, poor disability status—is less than 50% (44, 45). Therefore, our finding of HR of 1.07 for the development of poor disability status with a CRC diagnosis is comparable in magnitude to the risk ratio of 1.07 found by Braun et al., who observed a small increase in chair rise time to disability onset (43).

Furthermore, our findings aligned with another study demonstrating that survivors of CRC exhibited a higher prevalence of disability compared to individuals without CRC (22). Given the substantial costs of disabilities (46) and their impact on quality of life, as well as potentially secondary effects (47), it is critical to identify cancer survivors at risk of developing disability early, even with a minor hazard. Early rehabilitation has been shown to improve clinical outcomes and health-related quality of life (48).

Moreover, our findings regarding the significant factors associated with the development of poor disability status are in alignment with several studies of survivors of CRC and disability pension (49, 50). Older age, identifying as female, and having a higher cancer stage were all associated with the development of poor disability status and disability pension (49, 50). Of note, in our assessment of cancer-related treatments in our CRC survivor cohort, chemotherapy was not found to be a risk factor for the development of poor disability status, which is consistent with the study by Chen et al. (49) on the use of postoperative chemotherapy in predicting disability pension, whereas our identifying radiation therapy as a risk factor for the development of poor disability status is inconsistent with the findings of Chen et al. (50), whereby preoperative (chemo)radiotherapy was not significantly associated with disability pension. This difference could be due to a lack of distinction between preoperative and postoperative cancer-related treatments in our data (51, 52). To refine our findings in the future, a linkage is needed for more detailed treatment information, which may improve our model.

Our identification of race/ethnicity and the number of comorbid conditions as risk factors for disability align with previous studies of cancer survivors, including survivors of CRC (20, 53–55). Similar to Hewitt et al. and Okoro et al., we found that Hispanic or non-Hispanic Black survivors of CRC were more likely to develop poor disability status compared with non-Hispanic White survivors of CRC (20, 55). In addition, similar to Hewitt et al., Short et al., and Hung et al., we found that having one or more chronic conditions was significantly associated with poor disability status among survivors of CRC (20, 53, 54). The finding of greater risk in the Hispanic and Black survivor groups suggests that further investigation into the reasons for this greater risk for these two racial/ethnic groups is needed. Studies by Flores et al. and Odonkor et al. indicate that such differences could be due to the disparities in accessing healthcare services, including prevention, treatment, and rehabilitation, among the Hispanic or Black populations (56, 57). Our findings also suggest that data on social determinants to health (e.g., housing instability, food insecurity, transportation problems, utility help needs, interpersonal safety, family, community support) need to be collected to determine the domains associated with the development of poor disability status (58). In this way, targeted interventions can be developed to prevent or reduce future disabilities among cancer survivors.

Finally, our findings suggest that using Davidoff’s method (25) with administrative data is a potential tool for estimating the development of poor disability status among survivors of CRC at the population level. Notably, administrative data could potentially be used to identify high-risk groups in other cancer survivor populations at risk of poor disability status so that additional data, such as the social determinants of health, can be gathered to facilitate the development of disability prevention strategies.

The strengths of this study of cancer survivors and the development of poor disability status throughout the cancer care continuum include the study’s large sample size of survivors of CRC without preexisting functional impairments at Time 0, drawn from an easily accessible administrative database; a long follow-up period of up to 9 years; and an age-sex-matched non-cancer cohort. Despite these strengths, there are several limitations, as discussed below.

Limitations to our study include findings that differ from those of a similar study (49) on the risk factors (chemotherapy and radiation therapy) for the development of poor disability status for the survivors of CRC, indicating that administrative data may not have been sufficient or comprehensive. For example, our administrative data can identify only whether survivors of CRC had any cancer-related treatment, but knowing whether the treatment is preoperative or postoperative could be useful to assess future disabilities for survivors of CRC (51, 52). Additionally, the CRC diagnosis had a slight association (HR = 1.07) with developing poor disability status in the matched cancer and non-cancer cohorts, after adjusting for age, sex, race, income, education, and comorbidity. However, this correlation may reach null if further covariates are identified and managed within the model.

Another limitation is related to the disability status generated using Davidoff’s method. This method primarily targets general Medicare beneficiaries (25) and its predictive reliability has not been specifically validated for the CRC population. Furthermore, disability status was determined after the cancer diagnosis, and 90% of the CRC survivors in our study underwent surgery during the first year. Therefore, some temporary effects from the CRC surgery could have increased the probability of claim-based disability. Moreover, poor disability status could be a recurrent event. However, our study only focused on the first poor disability event after the cancer diagnosis. Therefore, future study is recommended to investigate the trajectory of poor disability status to further our understanding of changes in disability status and its implication on cancer survivors’ psychological distress, quality of life, and mortality.

The last limitation of our study is a limited generalizability. The study population is limited to Texas and Medicare fee-for-service beneficiaries. Therefore, our findings may not be applicable to study populations outside the Texas region. Moreover, the cancer treatment covariates from our administrative data are limited to those covered by Medicare, which may cause our findings to differ from studies that do not use Medicare data. For this reason, future studies are needed to test this method in different settings or in a national sample to verify our findings, which would allow for verification and a broader understanding of the implications beyond our current study population.

## 5 Conclusions

Overall, our results suggest that a CRC diagnosis may be a potential independent risk factor for the development of poor disability status in TCR-Medicare survivors of CRC. Although our hazard ratio of 1.07 is small, the marginal rise in the risk of developing poor disability status provides valuable insights for clinical providers concerning the potential contribution of the CRC diagnosis among vulnerable older survivors of CRC. Recognizing the immense cost of disabilities (46) and their negative impact on quality of life, as well as future secondary effects (47) underscores the critical importance of identifying cancer survivors at risk of developing disability early, even at a small risk. This proactive approach can facilitate early rehabilitation, which has been shown to enhance clinical outcomes, mitigate the negative impact on health-related quality of life, and reduce the overall cost of disabilities (48, 59, 60).

Specifically, for survivors of CRC, older age, female sex, identification as Hispanic or Black, having regional or distant disease at diagnosis, and having comorbidities were associated with a high risk of developing poor disability status. Particularly, older age, female sex, and identification as Hispanic or Black remained significant risk factors for survivors of CRC when compared to the non-cancer cohort. Furthermore, undergoing surgery or radiation therapy as cancer treatments emerged as risk factors for developing poor disability status. Together, these risk factors identified potential groups of survivors of CRC at risk for developing future poor disability status. Further research is warranted to develop targeted interventions aimed at reducing the risk of developing poor disability status, given the association between poor disability status and psychological distress, poorer quality of life, and mortality.

## Data availability statement

Data cannot be shared publicly because Medicare claims data release is controlled by a data user agreement.

## Ethics statement

Medicare claim database is a de-identified secondary data and the data release is controlled by a data user agreement. The consent of the study subject is not applicable. The University of Texas Medical Branch Institutional Review Board (IRB) deemed this study as exempt from IRB review.

## Author contributions

SZ: Writing – original draft, Writing – review & editing. L-NC: Project administration, Writing – review & editing, Formal Analysis, Software, Methodology, Writing – original draft, Data curation. MDS: Formal Analysis, Supervision, Writing – review & editing, Writing – original draft. HM: Writing – review & editing, Conceptualization, Supervision. JG: Writing – review & editing, Conceptualization, Data curation, Funding acquisition, Methodology, Resources, Supervision. Y-FK: Writing – review & editing, Conceptualization, Data curation, Formal Analysis, Methodology, Validation. SG: Data curation, Writing – review & editing, Supervision. CT: Writing – review & editing, Investigation. KB-E: Writing – review & editing, Investigation. EL: Writing – review & editing, Investigation. BD: Writing – review & editing, Investigation. SP: Writing – review & editing, Investigation. TC: Writing – review & editing. MCS: Conceptualization, Funding acquisition, Investigation, Project administration, Supervision, Writing – review & editing, Resources, Validation, Methodology, Writing – original draft.
