# Supplementary material for: Association of cancer diagnosis with disability status among older survivors of colorectal cancer: a population-based retrospective cohort study
Source: Front Oncol. 2024 Mar 15;14:1283252. doi: 10.3389/fonc.2024.1283252 (PMC10978737; doi:10.3389/fonc.2024.1283252)
Supplement: Supplementary file 1 [file Table_1.docx]

**Supplementary Materials**

**Table S1. Diagnosis and Procedure Code to Determine Cancer-Related Treatment**

| Treatment Type | ICD-9-CM | Revenue Center Code | Current Procedural Terminology (CPT) codes |
| --- | --- | --- | --- |
| Chemotherapy | Diagnostic code:  V581, V662, V672  Procedure code:  9925 | 0331, 0332, 0335 | 96400-96549, J9000-J9999, Q0083-Q0085 |
| Radiotherapy | Diagnostic code:  V580, V661, V671  Procedure code:  9221-9229 | 0330, 0333 |  |
| Surgery | Procedure code:  457, 458 |  | 44140, 44141, 44143, 44144, 44145, 44146, 44147, 44150, 44151, 44152, 44153, 44204, 44205, 44206, 44207, 44208, 44155, 44156, 44157, 44158, 45110, 45111, 45112, 45113, 45114, 45119, 45120, 45121, 45170, 45171, 45172, 44210, 44211, 44212, 44160, 44210, 45116, 45123, 45126, 45160, 45395, 45397, 45383, 45384, 45385, 4571, 4572, 4573, 4574, 4575, 4576, 4581, 4582, 4583, 1731, 1732, 1733, 1734, 1735, 1736, 4841, 4842, 4843, 4849, 4850, 4851, 4852, 4859, 4860, 4861, 4862, 4863, 4864, 4865, 4579, 1739, 4869 |

**Supplementary Table S2. Multivariable Cox Regression Modeling Analysis of Disability after Cancer Diagnosis among Survivors of Colorectal Cancer**

| Variable | Category | Adjusted HR (95% CI) | |
| --- | --- | --- | --- |
|  |  | Baseline treatment | Time-dependent treatment |
| Age, years | Reference: ≤ 70 |  |  |
|  | 71–75 | 1.36 (1.23–1.50)* | 1.35 (1.22–1.49)* |
|  | 76–80 | 1.94 (1.76–2.14)* | 1.93 (1.75–2.13)* |
|  | > 80 | 3.50 (3.19–3.83)* | 3.52 (3.22–3.85)* |
| Female |  | 1.50 (1.41–1.60)* | 1.50 (1.41–1.60)* |
| Origin and race^‡^ | Reference: White |  |  |
|  | Hispanic | 1.34 (1.22–1.46)* | 1.36 (1.24–1.48)* |
|  | Black | 1.21 (1.07–1.36)* | 1.22 (1.09–1.38)* |
| Income^§^ | Reference: Q1 (low) |  |  |
|  | Q2 | 0.98 (0.90–1.07) | 0.98 (0.90–1.07) |
|  | Q3 | 0.94 (0.85–1.04) | 0.93 (0.85–1.03) |
|  | Q4 (high) | 0.98 (0.86–1.11) | 0.98 (0.86–1.11) |
| Education^§^ | Reference: Q1 (low) |  |  |
|  | Q2 | 1.00 (0.92–1.09) | 1.00 (0.92–1.10) |
|  | Q3 | 0.94 (0.84–1.04) | 0.94 (0.85–1.04) |
|  | Q4 (high) | 0.87 (0.76–0.99)* | 0.87 (0.76–0.99)* |
| Comorbidity | Reference: No comorbidity |  |  |
|  | One comorbidity | 1.42 (1.32–1.53)* | 1.41 (1.31–1.52)* |
|  | More than one comorbidity | 2.18 (2.02–2.35)* | 2.16 (2.00–2.32)* |
| Stage | Reference: In situ |  |  |
|  | Localized | 1.08 (0.91–1.29) | 1.07 (0.90–1.27) |
|  | Regional | 1.43 (1.20–1.71)* | 1.39 (1.16–1.65)* |
|  | Distant | 2.26 (1.85–2.76)* | 2.25 (1.85–2.73)* |
| Treatment |  |  |  |
| Surgery |  | 0.72 (0.65–0.80)* | 0.96 (0.90–1.03) |
| Radiation |  | 1.21 (1.10–1.33)* | 1.22 (1.06–1.42)* |
| Chemotherapy |  | 0.93 (0.86–1.01) | 1.00 (0.85–1.16) |

^*^p<0.05

^‡^ Origin recode NHIA was applied to define the Hispanic group; subjects with unknown or other were not included.

^§^ Patients with unknown values were not included.

**Supplementary Table S3. Multivariable Marginal Cox Regression Modeling Analysis of Disability after Cancer Diagnosis (Index Date for Non-Cancer) for the Matched Cancer and Non-Cancer Cohorts**

| Variable | Category | Adjusted HR (95% CI) |
| --- | --- | --- |
| Cancer |  | 1.07 (1.02–1.13)* |
| Age, years | Reference: ≤ 70 |  |
|  | 71–75 | 1.36 (1.26–1.46)* |
|  | 76–80 | 1.98 (1.85–2.13)* |
|  | > 80 | 3.48 (3.25–3.72)* |
| Female sex |  | 1.51 (1.45–1.59)* |
| Race^‡^ | Reference: White |  |
|  | Hispanic | 1.37 (1.24–1.52)* |
|  | Black | 1.24 (1.14–1.36)* |
| Income | Reference: Q1 (low) |  |
|  | Q2 | 0.94 (0.88–1.00)* |
|  | Q3 | 0.91 (0.85–0.98)* |
|  | Q4 (high) | 1.01 (0.92–1.10) |
| Education | Reference: Q1 (low) |  |
|  | Q2 | 0.93 (0.88–0.99)* |
|  | Q3 | 0.87 (0.81–0.94)* |
|  | Q4 (high) | 0.79 (0.72–0.87)* |
| Comorbidity | Reference: No comorbidity |  |
|  | One comorbidity | 1.60 (1.51–1.69)* |
|  | More than one comorbidity | 2.37 (2.22–2.52)* |

^*^p<0.05.

^‡^ Medicare race; subjects with other race or unknown race were not included
